# Special Issue “Recent Progress in Hepatitis A Virus Research”

**DOI:** 10.3390/v14061138

**Published:** 2022-05-25

**Authors:** Sébastien Lhomme

**Affiliations:** 1 Virology Laboratory, Hôpital Purpan, CHU Toulouse, 31300 Toulouse, France; lhomme.s@chu-toulouse.fr; 2 Institut Toulousain des Maladies Infectieuses et Inflammatoires (Infinity), INSERM UMR1291-CNRS UMR5051, 31300 Toulouse, France; 3 Université Toulouse III Paul-Sabatier, 31062 Toulouse, France

The hepatitis A virus (HAV) is still one of the leading causes of acute viral hepatitis worldwide, despite there being an anti-HAV vaccine. It is a virus of concern not only because of its global distribution but also because of its quite unique quasi-enveloped form [1].

We still need biological diagnosis because hepatitis A is clinically indistinguishable from other viral causes of hepatitis and validated home-made assays are still good alternatives to commercial assays where or when commercial are difficult to obtain. Kozak et al. tested and validated a home-made assay on both blood and stool samples [2]. Genotyping methods are also needed to study the emergence and spread of particular strains and to investigate clusters. Studies of VP1/2A sequences (267 bp) and complete VP1 sequences (953 bp) showed that the HAV strains circulating in Brazil belong to subgenotype IA and that most of the strains were closely related to that isolated in outbreaks that occurred in several European countries in 2016 [3]. Honda et al. also described a recent male-dominant hepatitis A outbreak in Japan involving subgenotype IA. The genotype was determined by sequencing part (168 bp) of the VP1-2A region [4].

Environmental surveillance can also help provide a more complete picture of strains circulating in a community [5], such as the spread of a particular strain during an outbreak. The contents of treated wastewater discharges can also be examined to identify the risk of exposure of communities using water downstream from wastewater treatment plants [6]. Analysis of clinical, food, and environmental data is in agreement with the One Health approach, and should increase in the next few years.

The review written by Migueres et al. summarizes the epidemiology of HAV infection, the main groups of people at risk and the strategies used to prevent infections [7]. It also provides an update on the development of antiviral drugs targeting HAV. These would be a great step forward for treating severe and fulminant forms. It was widely accepted that liver injuries were due to an adaptive immune response, especially that of HAV-specific cytotoxic T cells, as the replication of HAV is noncytopathic in vitro. However, the review by Wang et al., which summarizes the current knowledge of the mechanisms of hepatocellular injury, suggests that innate cells like natural killer (NK) and NKT cells are involved [8].

The 5 original research paper and 2 tremendous reviews in this issue will, I am sure, contribute to our knowledge of the hepatitis A virus. I thank all the contributors who made this special issue of “Recent Progress in Hepatitis A Virus Research” possible.
